# Hearing loss in HEMS operations: a prospective analysis of audiometric tests for temporary hearing threshold shift of an air rescue helicopter crew during several days of emergency duty

**DOI:** 10.1186/s12245-026-01382-z

**Published:** 2026-09-21

**Authors:** Michelle Hohm, Harald Schulze, Sandra Schmidt, Dennis Matthias Ritter, Willi Schmidbauer, Jens Schwietring, Felix Carl Fabian Schmitt, Christoph Walter Jaenig, Tobias Gruebl

**Affiliations:** 1 Hannover Medical School, Hannover, Germany; 2 Department of Occupational Medicine, Bundeswehr Central Hospital, Koblenz, Germany; 3 Department of Otorhinolaryngology, Bundeswehr Central Hospital, Koblenz, Germany; 4 Department of Otorhinolaryngology, University Medical Center of the Johannes Gutenberg University, Mainz, Germany; 5 Medical Service Command, Bundeswehr, Koblenz, Germany; 6 Department of Anaesthesiology, Intensive Care Medicine, Emergency Medicine, and Pain Therapy, Bundeswehr Central Hospital, Koblenz, Germany; 7 Department of Medicine, ADAC Air Rescue Non Profit Limited liability Company, Muenchen, Germany; 8 Department of Anesthesiology and Intensive Care Medicine, Philipps-University, Baldingerstraße, Marburg, 35033 Germany

**Keywords:** Air rescue, Helicopter emergency medical services, Noise, Hearing protection, Audiometry

## Abstract

**Background:**

Helicopter Emergency Medical (HEMS) crews are exposed to varying levels of noise at work. Noise levels regularly exceed 85 dB(A) and can be as high as 108 dB(A). Noise exposure can reduce concentration and cause hearing damage. The present study was conducted to assess objective Hearing sensitivity shift of air rescue crews.

**Methods:**

Audiometric data of HEMS crew of an Airbus EC 135 were collected during two months of duty at frequencies from 500 to 8000 Hz. Audiometric tests were performed at the beginning and end of a shift and, if possible, thirty minutes after every HEMS operation. Crew members were asked to complete a questionnaire before every measurement on ringing in the ears and exposure to elevated noise levels without hearing protection. Results of the audiometric measurements were analysed using linear mixed models for the whole group and for subgroups of pilots and medical personnel with and without additional hearing protection.

**Results:**

On 17 days with a median daily number of 6 HEMS flights, a total of *n* = 236 audiometric tests were conducted for 18 crew members, of whom four (22%) were pilots and fourteen (78%) were medical personnel. The mean noise exposure duration was 169.9 (± 49.2) minutes. The mean flight duration was 50 (± 40.2) minutes. Across all subgroups, subjects were able to hear a test tone at a mean level of 8.2 (± 11.1) dB(A) at the beginning of a shift and 11.7 (± 12.1) dB(A) at the end of a shift. The initial difference of hearing threshold between the left and the right ear was evened out over the work shift (beginning β = 2.9 *p* < 0,001vs. after noise exposure 0.2, *p* = 0.434). Hearing threshold shift was greater in medical personnel who wore no additional hearing protection (6.8 dB(A)) than in pilots (1.9 dB(A)) and medical personnel who used additional hearing protection (0.5 dB(A)). The greatest hearing threshold level occurred at frequencies between 4000 and 8000 Hz.

**Conclusions:**

Hearing sensitivity of HEMS crews substantially declines over the course of a shift. Unprotected medical personnel seems more affected than pilots. Hearing threshold level is greater in left ears than in right ears and most severely affects the ability to hear high-frequency sounds. Helmets alone may not provide adequate hearing protection.

**Supplementary Information:**

The online version contains supplementary material available at https://doi.org/10.1186/s12245-026-01382-z.

## Introduction

During helicopter emergency medical services (HEMS) operations, medical personnel are sometimes exposed to rotor and engine noise outside the helicopter without wearing a helmet with or single hearing protection. This may be the case during take-off or landing, when helicopter engines are running and medical personnel, such as emergency physicians and helicopter emergency medical services technical crew members (HEMS TCs), approach or depart the helicopter in order to reach a patient quickly [1]. The types of helicopters (EC 135, H 145) that are commonly used by HEMS generate noise levels of approximately 86 dB(A) during flights and peak noise levels of 107.8 dB(A) during take-off and landing, during winch operations, and during refueling [2–5]. 

Exposure to high noise levels depending on extent, duration, and frequency causes hearing damage and especially affects the ability to hear high-frequency sounds [6–8]. Hearing protection devices must be used to comply with old but still applicable occupational health and safety regulations. They are in particular recommended for tasks that are intellectually demanding or require high levels of concentration and are associated with prolonged exposure to noise levels of at least 54 dB(A). In such noise situations, hearing protection helps prevent difficulty concentrating, balance problems, and headaches [9–13]. 

The use of earmuffs that are attached to a helmet is recommended during flights. Hearing protectors as defined in the DIN EN 352:1–3 classification of hearing protection devices should be worn outside the helicopter [14]. 

Several studies have shown that HEMS operations could associated with elevated levels of noise that should be appropriately addressed [15, 16]. Moreover, it is possible that the helmets with earmuffs currently used provide insufficient noise attenuation [17, 18]. This can lead to long-term damage, frequently in the left ear [3, 15–18]. 

This study investigates changes in hearing threshold levels in air rescue helicopter crews during a normal shift.

## Methods

**Fig. 1:**
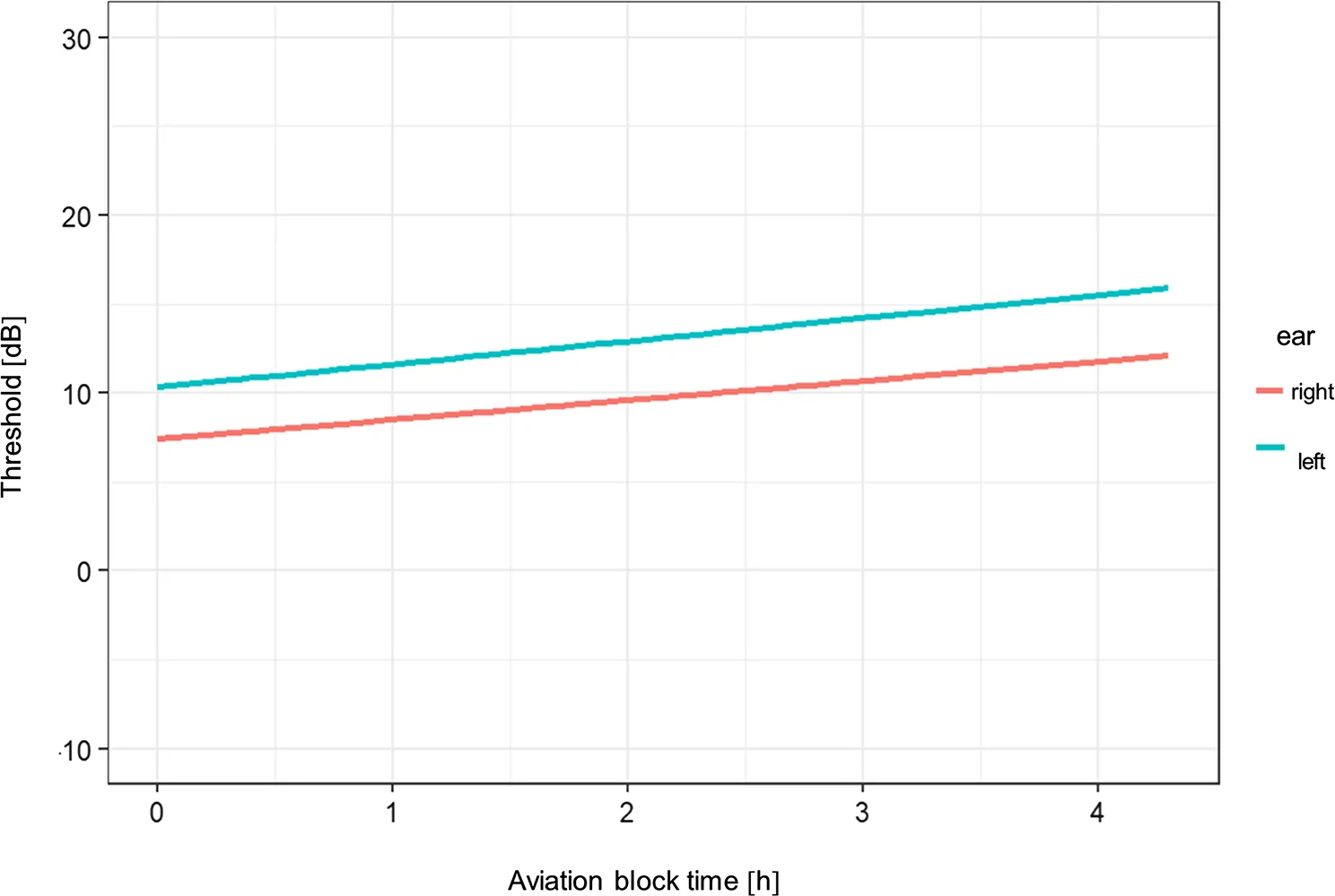
Hearing threshold shift after extended exposure to noise

**Fig. 2:**
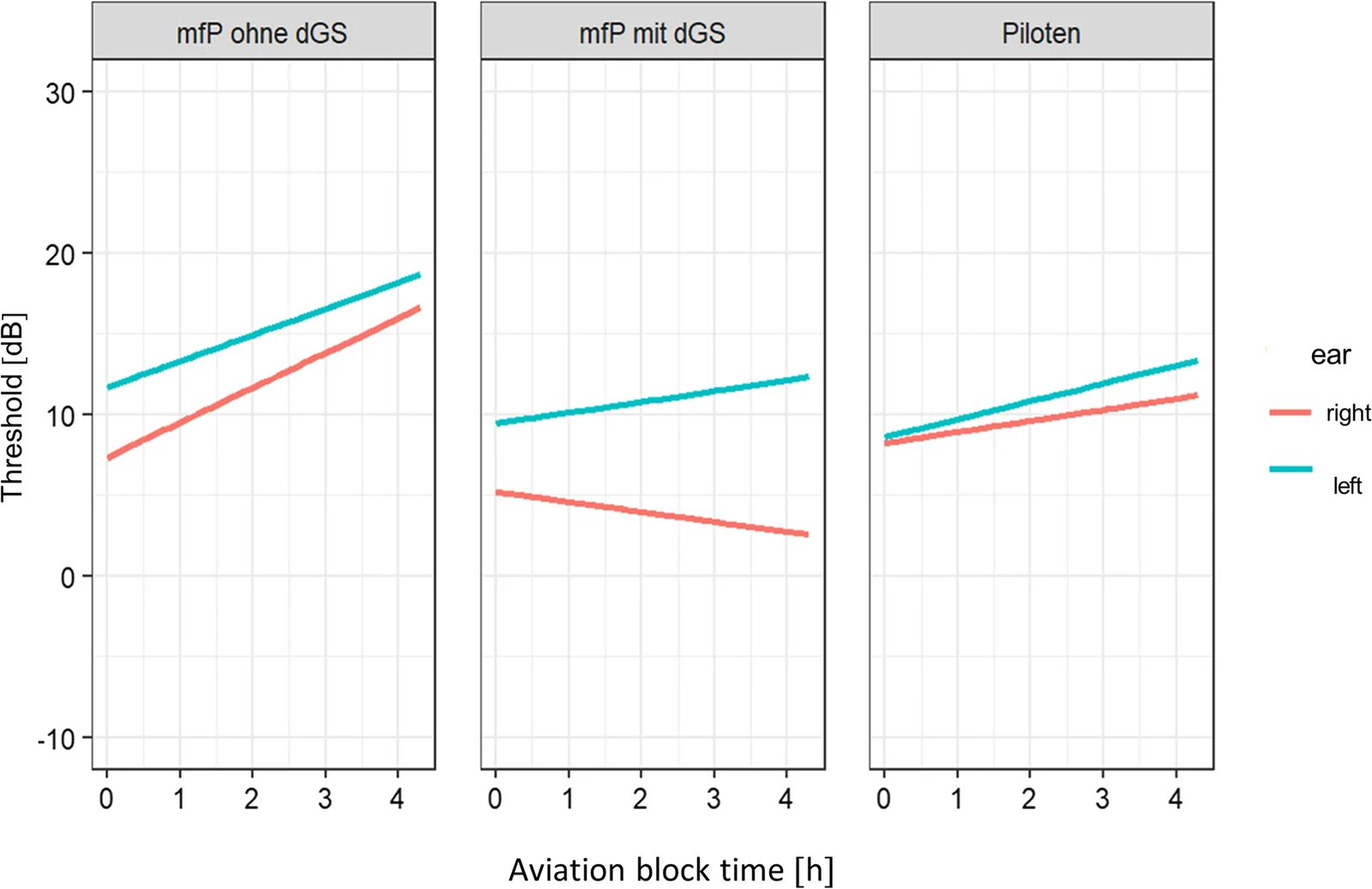
Hearing threshold shift after extended exposure to noise in subgroups (medCrew = medical personal, HearProt = hearing protection device, addHearProt = additional hearing protection device)

Following approval from the ethics committee (Ref.: 2019–14629, Medical Association of Rhineland-Palatinate), audiometric tests were performed on different HEMS crews at an air rescue station between August and September 2019. A air rescue work shift includes opening the station and checking the equipment, carrying out HEMS missions until sunset, packing up the equipment, and leaving the station again. Audiograms were produced using the audiometric unit ear3.0, Auritec, Hamburg, Germany with air-conduction Headphones AT1350, standard Response-Button and annual system calibration (Add Fig. 1). Test tones between − 10 dB(A) and a maximum of 80 dB(A) at frequencies from 500 to 8000 Hz were presented manually ascending in 5 dB increments, two times in alternating order to the subjects (Hughson-Westlake methodology), who were asked to acknowledge hearing the tones by pressing on the response button. This method was used to determine the lowest audible intensity (dB(A)) per frequency (Hz). All audiometric tests were performed in the samea closed and secluded room without ambient noise at the beginning of a shift, at the end of a shift, and generally thirty minutes after every HEMS operation if the helicopter crew returned to the air rescue station. In rare exceptions, the 30 min could not be met because medical personnel were needed in the emergency room immediately after landing. These measurements were included, nonetheless. The order in which the personnel were tested was different every time. Prior to every test, subjects were asked to complete a questionnaire in order to provide data on ringing in the ears, hearing threshold shift, the use of hearing protectors in addition to a helmet, pre-existing hearing conditions, and exposure to any noise without hearing protection (Add. Figure 2). There were no exclusion criteria for participation in the study. All employees who reported for duty were eligible for inclusion in the study. Tests were performed on the basis of the preventive occupational health examination for noise-exposed workers (G-20) [19]. Tests on the frequencies 500 Hz and 1000 Hz were conducted twice in order to aid familiarization. If the measurement results for individual frequencies differed significantly from others, a control measurement was performed at the specific frequency in the same test session. The measurement was performed by a study assistant. The participants could not see the audiometry device or the noise exposure level (blinded). Anonymization was achieved by each test person independently coming up with a personal code, which they noted on the audiometry sheets and questionnaires, so that only the person themselves can identify their data. Statistical analysis of audiometric measurements was performed using linear mixed models (LMM). Multiple testing of the same participants are taken into account here and evaluated as a cluster effect. The dependent variable consisted of the lowest sound intensity in decibels (dB(A)) that a subject reported being able to perceive. The independent variable was the time that a subject had been exposed to helicopter noise during a shift before audiometric testing was performed. Cluster effects due to individual subject reporting and due to the influence of sound frequency on the hearing threshold shift were modelled as random effects. In addition, we defined the group of subjects, and interactions with noise exposure duration as fixed effects to compare subgroups and ear side in terms of hearing threshold shift. Regression coefficients, 95% confidence intervals, and p values (t-test) are provided in order to present the effects [20]. The level of significance was set at 5%. For descriptive analyses of audiometric data and the group of subjects, “means ± standard deviations” were used for continuous variables, “medians” for count data, and “n (%)” for absolute numbers and percentages. All data were analysed using R software, version 4.1.2 (Imer and ImerTest packages). Independently of the study, some crew members wear additional earmolds protectors or earplugs. Medical personnel who use hearing protectors in addition to a helmet wear double hearing protection during flights. Depending on the type of hearing protection and the frequency, noise can be further reduced by up to 41 dB(A) at a frequency of 2000 Hz [21]. The mean reduction that is achieved using earmolds is 12–24 dB(A) at a frequency of 4000 Hz [22, 23]. Changes in the sensitivity of frequencies on the basis of audiometric measurements on HEMS crew members were the primary endpoint in this study. Differences between subgroups of crew members were a secondary endpoint. Unlike pilots, medical personnel with or without additional hearing protection (medCrew normal HearProt/ medCrew addHearProt) sit in a helicopter with their left ear to the window (one facing forward on left side in the helicopter, one backward on the right). The volume of the onboard intercom system in the used air rescue helicopter could not be adjusted individually.

## Results

A total of *n* = 236 audiometric tests were performed on 18 subjects in 17 duty days. The group of subjects consisted of *n* = 4 pilots, *n* = 4 HEMS TCs, *n* = 8 physicians, and *n* = 2 trainees. A comparison of the results of the audiometric tests that were performed at the beginning of a work shift and those that were obtained at the end of a shift shows that hearing ability tended to decline in all three subgroups of subjects over the course of a shift (Table 1). The hearing threshold shift increased by a mean of 3.5 dB(A). Medical personnel without additional hearing protection were affected the most. In this subgroup, the hearing threshold shift increased by a mean of 6.8 dB(A).

**Table 1:** Descriptive comparison of subgroups

	Group			Total group
	Medical personnel without additional hearing protection	Medical personnel with additional hearing protection	Pilots	
n (%)	10 (55.56%)	4 (22.22%)	4 (22.22%)	18 (100%)
Number of audiometric tests	118 (50%)	44 (18.64%)	74 (31.36%)	236 (100%)
At the beginning of a shift [dB(A)]	7.7 (± 10.3)	9.5 (± 12.7)	8.2 (± 10.9)	8.2 (± 11.1)
At the end of a shift [dB(A)]	14.5 (± 13.4)	10 (± 11.5)	10.1 (± 10.5)	11.7 (± 12.1)
Hearing threshold shift [dB(A)]	6.8	0.5	1.9	3.5
Noise exposure per shift [minutes]	175.7 (± 30.9)	177.6 (± 52.7)	158.2 (± 62.2)	169.9 (± 49.2)
Number of EMS operations per shift	6 (5 to 7.25)	5 (4 to 7)	6 (5 to 7)	6 (5 to 7)
Duration of an EMS operation [minutes]	48.3 (± 36.1)	53 (± 47.2)	51.1 (± 41.9)	50 (± 40.2)

Results are expressed as numbers (n) and percentages (%), means (± standard deviations), or medians (first to third quartiles)

Table 2 shows the mean sound intensities perceived by the whole group of subjects at different frequencies during a shift.

**Table 2:** Hearing threshold over the course of a work shift at different frequencies

	Frequency						
	500 Hz	1000 Hz	2000 Hz	3000 Hz	4000 Hz	6000 Hz	8000 Hz
At the beginning of a shift	1.6 (± 6.3)	3.7 (± 5.7)	2.5 (± 5.4)	10 (± 11.8)	13.3 (± 12.8)	13.8 (± 12.2)	12.5 (± 11.4)
During a shift	4.6 (± 8.3)	6.8 (± 7)	4.4 (± 6.6)	13.8 (± 13.2)	16.8 (± 13)	18.7 (± 12.6)	17.9 (± 14.4)
At the end of a shift	6.4 (± 9.3)	7.5 (± 6.4	5.3 (± 8)	12.7 (± 12.4)	16.5 (± 13.4)	17.7 (± 11.9)	16 (± 14.7)
Recovery after the end of a shift	4.2 (± 5.4)	6.7 (± 4.4)	4.2 (± 4.6)	8.8 (± 8.6)	14.2 (± 13.8)	21.7 (± 18.1)	20.5 (± 20.7)

Results are expressed in dB(A) as means (± standard deviations)

Higher values indicate a decline in the subjects’ ability to hear

The results indicate two trends.

- The hearing threshold shift increased at higher frequencies. The highest hearing threshold shift was measured at a frequency of 8000 Hz. At the same time, hearing thresholds shift were more widely scattered at higher frequencies.
- The hearing threshold shift increased at all frequencies over the course of a work shift. At lower frequencies (from 500 to 2000 Hz), the highest mean thresholds shift were observed at the end of a work shift. At frequencies of 3000 Hz and above, the highest mean thresholds shift were measured during a work shift. The audiometric tests that were performed after the end of a work shift showed some degree of recovery at frequencies from 500 to 4000 Hz and the highest absolute hearing thresholds shift at the highest frequencies (6000 and 8000 Hz).

The results of regression analysis are shown in Figs. 1 and 2.

In the whole group of subjects, the hearing threshold shift increased by all subjects and in a mean of 1.19 dB(A) with every additional hour of noise exposure. This hourly increase was statistically significant (*p* < 0.001, Table 3). In addition, Fig. 1 shows that hearing threshold level in the left ear tended to be greater than in the right ear. Extended noise exposure, however, led to hearing threshold shift in both ears (*p* = 0.001 for right ears and *p* < 0.001 for left ears, Table 3). There was only a minor difference between the two sides (*p* = 0.434, Table 5).

**Table 3:** Linear mixed model: hearing threshold shift by subgroups

Group	Ear	Regression coefficient [beta]	95% confidence interval	*P* value
Medical personnel without additional hearing protection	Both	1.89	(1.33 to 2.45)	**< 0.001**
	Left	1.63	(0.96 to 2.3)	**< 0.001**
	Right	2.15	(1.49 to 2.82)	**< 0.001**
Medical personnel with additional hearing protection	Both	0.02	(-0.83 to 0.88)	0.953
	Left	0.68	(-0.32 to 1.69)	0.18
	Right	-0.61	(-1.62 to 0.39)	0.226
Pilots	Both	0.9	(0.11 to 1.69)	**0.028**
	Left	1.11	(0.23 to 2)	**0.016**
	Right	0.69	(-0.2 to 1.58)	0.123
Total group	Both	1.19	(0.64 to 1.74)	**< 0.001**
	Left	1.3	(0.7 to 1.9)	**< 0.001**
	Right	1.09	(0.49 to 1.69)	**0.001**

The regression coefficient represents the mean increase in the hearing threshold shift that was observed with every additional hour of noise exposure

A higher regression coefficient indicates a greater hearing threshold

**Table 4:** Linear mixed model: comparison of left and right ears

Group	Comparison of	Regression coefficient [beta]	95% confidence interval	*P* value
Medical personnel without additional hearing protection	Measurements at the beginning of a shift	4.31	(2.87 to 5.76)	**< 0.001**
	Hearing threshold after noise exposure	-0.53	(-1.3 to 0.24)	0.181
Medical personnel with additional hearing protection	Measurements at the beginning of a shift	4.22	(1.99 to 6.44)	**< 0.001**
	Hearing threshold after noise exposure	1.29	(0.16 to 2.43)	**0.026**
Pilots	Measurements at the beginning of a shift	0.37	(-1.34 to 2.08)	0.673
	Hearing threshold after noise exposure	0.42	(-0.5 to 1.34)	0.369
Total group	Measurements at the beginning of a shift	2.91	(1.91 to 3.9)	**< 0.001**
	Hearing threshold after noise exposure	0.21	(-0.32 to 0.74)	0.434

The regression coefficient represents the difference in hearing threshold shift between left and right ears

The higher the regression coefficient, the greater the hearing threshold shift in the left ear in comparison to the right ear

Moreover, medical personnel without additional hearing protection were affected the most. The hearing threshold shift increased by a mean of 1.89 dB(A) per hour (*p* < 0.001, Table 3). By contrast, this threshold shift increased by a mean of only 0.02 dB(A) per hour when hearing protection was worn continuously. This difference was significant (*p* < 0.001, Table 4). Pilots showed a mean increase in the lower threshold shift of 0.9 dB(A) per hour (*p* = 0.028, Table 3). The difference between this threshold shift and that measured for medical personnel without additional hearing protection was only just statistically significant (*p* = 0.042 in Table 4). Figure 2; Table 4 also reveal that the difference in hearing threshold level between the subgroups was greater for right ears than for left ears.

**Table 5:** Linear mixed model: comparison of hearing thresholde shift in different subgroups

Comparison	Ear	Regression coefficient [beta]	95% confidence interval	*P* value
Medical personnel with additional hearing protection versus medical personnel without additional hearing protection	Both	-1.87	(-2.88 to -0.86)	**< 0.001**
	Left	-0.95	(-2.14 to 0.25)	0.118
	Right	-2.77	(-3.96 to -1.57)	**< 0.001**
Pilots versus medical personnel without additional hearing protection	Both	-0.99	(-1.94 to -0.04)	**0.042**
	Left	-0.52	(-1.61 to 0.58)	0.344
	Right	-1.46	(-2.56 to -0.37)	**0.01**

The regression coefficient indicates the mean lower increase in the hearing threshold shift [dB(A)] that was observed with every additional hour of noise exposure in the subject group that was compared with the control group (medical personnel without additional hearing protection)

The lower the regression coefficient, the less severe the hearing threshold shift in the subject group that was compared with the control group

Unlike pilots, medical personnel showed significant differences in the hearing thresholds level for their right and left ears even at the beginning of their shift, when they had not yet been exposed to noise (Table 5).

An analysis of the questionnaires revealed that four subjects experienced ringing in the ears in the group with no hearing protection and the pilot group. No subject reported subjective higher or changed hearing threshold. Four subjects had a prior history of hearing damage (with an audiometric notch at 4000 Hz). No subject reported relevant noise exposure during the eight hours preceding the beginning of a shift. Four subjects used additional hearing protection, one of whom had a prior history of changed/ higher hearing threshold.

## Discussion

In the study presented here, hearing sensitivity in rescue helicopter crews significantly declined over the course of a shift. Hearing threshold level was greater in the left ear than in the right ear. A possible explanation may be the seating positions of HEMS TCs on the left front seat and the backwards seating position of the emergency physicians in the helicopter. Some differences between left and right ears, however, were detected in pilots too (Add. Figure 3). There are no anatomical or physiological differences between left and right ears which suggest that one ear may be more sensitive to noise than the other. Some other studies reported that left ears appeared to be more susceptible to hearing threshold shift than right ears, too [16, 17]. 

The greatest hearing threshold shift was detected in medical personnel, who are usually more often exposed to helicopter noise without wearing appropriate hearing protection.

These subjects showed generally better hearing than the other subjects. As at other air rescue stations, medical helicopter personnel are changed more often than pilots and thus perform a lower number of HEMS operations than pilots. While the number of missions per day remains the same, medical personnel fly fewer days at a time than pilots. Although medical personnel, compared to pilots, are briefly exposed to higher noise levels, the noise exposure of pilots can be longer over the course of a shift. The hearing ability of pilots ranged between that of the other two subgroups.

In all groups, the ability to hear high-frequency sounds was most severely compromised. This finding is consistent with the fact that noise affects in particular the ability to hear high- frequency sounds [6, 8]. 

The variability of the results for hearing threshold shift in response to continuous noise exposure considerably decreased in all groups over the course of a shift. There was a certain stronger effect affecting those with better hearing at the beginning of a shift.

The maximum permissible exposure to a noise level of 85 dB(A) is eight hours. The − 3dB halving principle is applied to higher noise levels. For example, the maximum duration of exposure to a noise level of 91 dB(A) is two hours, to 97 dB(A) thirty minutes, and to 106 dB(A) 3.8 min [9, 24, 25]. This means that the maximum duration of exposure to a peak noise level of 107.8 dB(A), which can occur the refueller during refueling outside the helicopter, should be less than 3.8 min and to a peak noise level of 104 dB(A), to which crew members are exposed when they approach or depart a helicopter with rotors running, should be less than 7.5 min per shift [5]. In addition, impulse noise, for example from securing the stretcher in the rear of the helicopter, is particularly harmful [26]. In order to address peak noise levels (> 90 dB), the operators of air rescue stations provide their personnel with additional earplugs.

Although the study presented here has some limitations, exposure to noise during flight without dual hearing protection can be expected to lead to hearing impairment. The group of subjects who were studied here was small. For this reason, it was not subdivided by sex, age or medical history. The focus of this study was on the deterioration of hearing over the course of a work shift and not on the absolute intensity of a sound that a subject can hear. This means that we addressed the development of hearing threshold shift from noise exposure independently of the subject’s normal hearing ability and age-related mean threshold shifts [19, 27, 28]. Sleep duration and the subjects’ fitness on the day of the tests were not included either. Furthermore, the number of consecutive exposure days varied. Exposure to noise over several consecutive days can result in a greater deterioration of hearing than when periods of exposure are interspersed with several days of recovery. A clearer interpretation of our findings might be possible if our results were compared with data on how the hearing sensitivity usually decreases over the course of a day in the absence of exposure to elevated noise levels.

It should also be noted that no actual noise measurements were taken for the helicopter used in this study. We based our calculations solely on the theoretical noise levels reported in the known literature. However, depending on the environment and the technical condition of the helicopter, different noise levels are possible. This could have a direct impact on the interpretation of the exposure-effect findings.Another limitation of the study is that it was not always possible to perform audiometric tests between noise exposures thirty minutes after landing, i.e. when there was no time between two HEMS operations or when medical crew members accompanied a patient to a hospital’s resuscitation room. This could lead to an examination of different points on the hearing recovery curve, ot ensuring that the real extent of hearing loss was detected. The earlier return of pilots to the hangar was also the reason for different numbers of tests per participant.

It is also worth mentioning that frequent audiometric tests conducted within a day could cause a learning effect among the participants. As a result, a shift in hearing thresholds - particularly in measurements taken later the day - may have been underestimated. This would be particularly relevant for medical personnel with a hearing threshold shift (6.8 dB) over the work shift. It should also be noted that, in multiple tests, a test-retest variance of +/- 5 dB should be assumed, which could shift the measured effects accordingly in either direction.Moreover, subjects used different types of additional hearing protection devices so that a general level of additional reduction (dB(A)) could not be determined in the study. The study is also limited by the lack of randomization since crew members decided on their own initiative and independently of the study whether or not to wear additional hearing protection and, if so, what type to use. As a result of this lack of randomization, the statistical analysis of the benefit of additional hearing protection is inaccurate.

Despite these limitations, we analysed the results of the study in order to obtain evidence on a possible deterioration of hearing over the course of a shift and on the possible benefits of additional hearing protection for crew members.

In this study, participants used three types of hearing protection, either alone or in combination. Each device has its advantages and disadvantages. For example earmuffs that are attached to helmets are easy to put on and take off, but lose their effectiveness when the helmets remain in the helicopter when egressing. Earplugs protect from continuous noise but can sometimes cause pain in the ear canal. Earmolds used as active hearing protection in combination earmuffs of the helmet provide individual hearing protection even after the helmet is removed and help crew members clearly understand the radio communication within the helicopter [12, 29, 30]. However, these are not yet provided as standard equipment, so the crew member must procure them on their own.

The fact that the change in hearing threshold was less severe in subjects with additional hearing protection is indicative of the positive effects of this type of hearing protection from the perspective of preventive medicine.

## Conclusions

The hearing sensitivity by rescue helicopter crews seems to decrease over the course of a shift. Although there are few studies identifying pilots or medical personnel as groups at risk of work-related hearing threshold shift, the results of the study presented here suggest the presence of hearing impairment in crew members. For this reason, crew members should wear hearing protection devices not only inside but also outside the helicopter when they are exposed to elevated noise levels. Since medical personnel often approach or depart a rescue helicopter while its engines are running, they should be advised to already wear hearing protection when they leave the rescue station and to not remove the devices until they arrive at the scene at a safe distance from the helicopter.

Noise-induced hearing threshold shift usually results from continuous exposure to noise over a period of approximately ten to fifteen years [31]. Although noise-induced hearing threshold shift is not always the result of occupational exposure, it is one of the most widely recognized and most common occupational diseases [12, 32, 33]. 

Further studies should be conducted to assess the validity of the data presented here. 

## Supplementary Information

Below is the link to the electronic supplementary material.

Supplementary Material 1

## Data Availability

The datasets used and/or analysed during the current study are available from the corresponding author on reasonable request.

## Abbreviations

ADAC: Allgemeiner deutscher automobil club (General German Automobile Club)
dB(A): decibel
HEMS: Helicopter emergency medical service
HEMS TC: Helicopter emergency medical service technical crew member
Hz: Hertz
LMM: Linear mixed model
