# Supplementary material for: Hearing loss in HEMS operations: a prospective analysis of audiometric tests for temporary hearing threshold shift of an air rescue helicopter crew during several days of emergency duty
Source: Int J Emerg Med. 2026 Sep 21;19:238. doi: 10.1186/s12245-026-01382-z (PMC13595511; doi:10.1186/s12245-026-01382-z)
Supplement: Supplementary file 1 [file 12245_2026_1382_MOESM1_ESM.docx]

Supplementary material

**Additional Figure 1:** Setup of the measuring unit at the air rescue station

**Q U E S T I O N N A I R E**

(First letter of first name - last letter of last name -

Day of birth - last digit of birth year (e.g., MN09 for

Max Mustermann, January 1, 2019)

Pseudonym:

Date:	Operation-No. today:

Time:	Aviation time (h/min):

Meas-No.:

shiftbeginn	after HEMS operation	shiftend

Yes	No

1. Have you been exposed to loud noise in the 8 hours before

your shift began?

2. Have you been standing near a helicopter with its engine running

today without wearing ear protection?

3. Are you currently experiencing hearing loss?

4. Are you currently experiencing ringing in your ears?

5. Did you use privately purchased in-ear hearing protection

(active or passive) throughout your shift today?

7. Do you have any pre-existing hearing problems?

If so, what kind?

_________________________________________________________________________

_________________________________________________________________________

_________________________________________________________________________

**Additional Figure 2:** Questionnaire presented to participants at each audiometric test

MD

HEMS

Pilot

**Additional Figure 3:** Seating positions in the air rescue helicopter
